# Mechanical circulatory support bridge for restrictive cardiomyopathy with biventricular and pulmonary failure to cardiac transplantation

**DOI:** 10.1007/s12055-024-01847-x

**Published:** 2024-10-28

**Authors:** Sandeep Sainathan, Leonardo Mulinari

**Affiliations:** Section of Pediatric Cardiothoracic Surgery, University of Miami, 90 SW 3rd Street, Suite 3503, Miami, FL 33130 USA

**Keywords:** Restrictive cardiomyopathy, Biventricular failure, Respiratory failure, Mechanical circulatory support

## Abstract

We describe a simplified technique for mechanical circulatory support using Berlin EXCOR cannulas in a toddler with restrictive cardiomyopathy and biventricular and respiratory failure as a successful bridge to cardiac transplantation.

## Introduction

Restrictive cardiomyopathy (RCM) can be challenging to mechanically support due to a small left ventricular cavity size [1, 2]. It can become further challenging when biventricular support is required, leading to poorer outcomes [1, 3]. In this case report, we describe a simplified technique for cardiopulmonary support in a patient with RCM with a successful bridge to cardiac transplantation.

## Case report

**Fig. 1:**
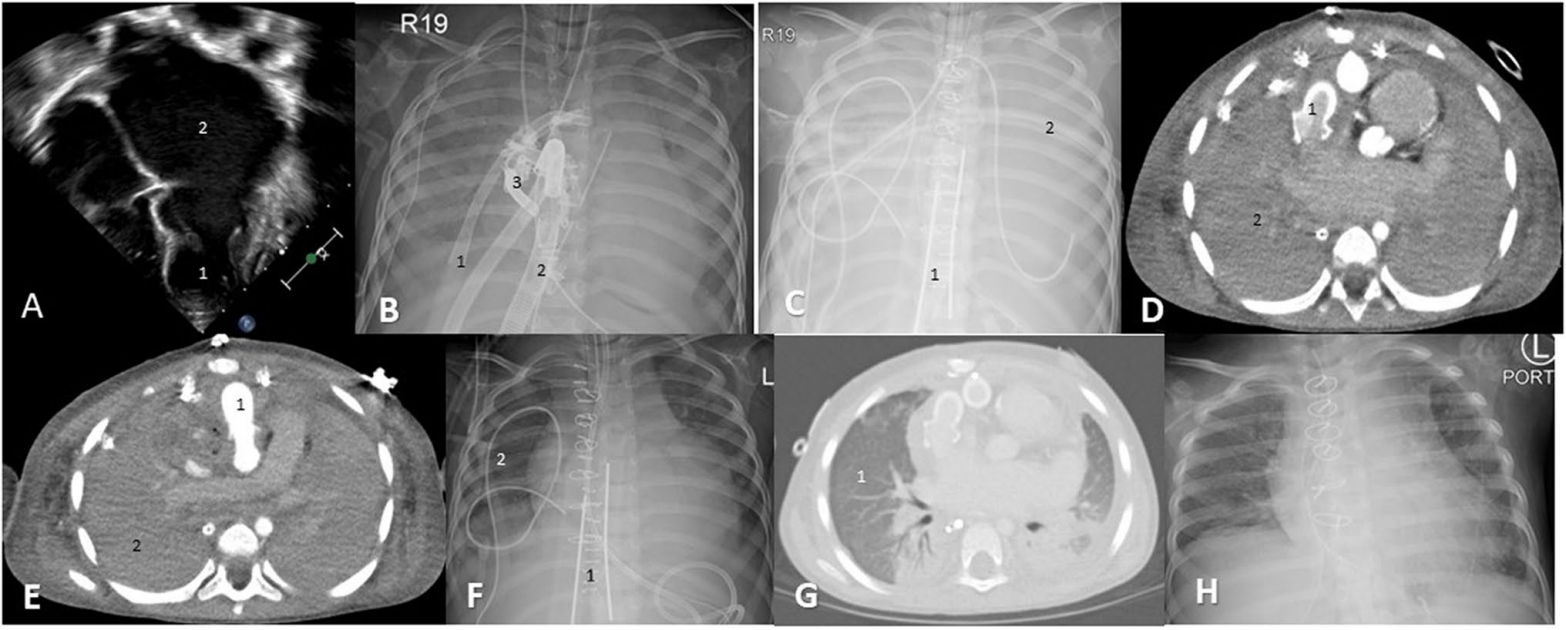
**A** Echocardiogram showing a non-dilated left ventricle [1] and severely dilated left atrium [2]. **B** Chest X-ray showing veno-arterial ECMO (Arterial cannula-1, venous cannula-2) with a left ventricular vent [3]. **C** Chest X-ray status post Berlin EXCOR cannula [1] insertion with veno-arterial ECMO and pulmonary hemorrhage [2]. **D** Computed tomography of the chest showing Berlin EXCOR aortic cannula with interposition graft [1] on the right atrial free wall for venous drainage and consolidated lungs from pulmonary hemorrhage [2]. **E** Computed tomography of the chest showing Berlin EXCOR aortic cannula with interposition graft [1] on the ascending aorta as outflow cannula and consolidated lungs from pulmonary hemorrhage [2]. **F** Chest X-ray showing Berlin EXCOR [1] veno-arterial ECMO with clearance of the pulmonary hemorrhage [2]. **G** Computed tomography of the chest showing resolution of pulmonary hemorrhage [1]. **H** Chest X-ray status post cardiac transplantation. ECMO, extracorporeal membrane oxygenation

**Fig. 2:**
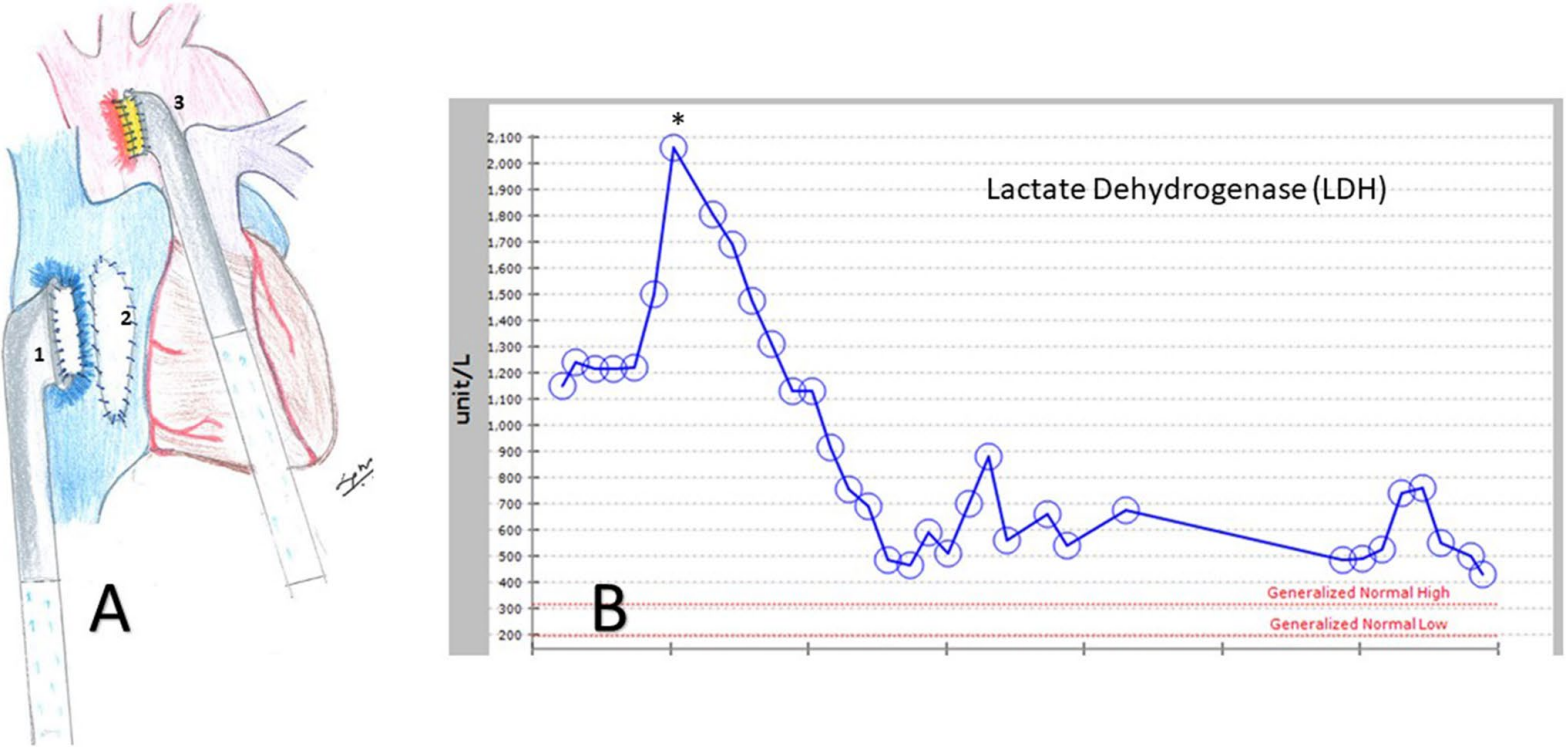
**A** Illustration showing a Berlin EXCOR aortic cannula with an interposition graft sutured to the right atrial free wall [1] as the inflow cannula, a surgical atrial septectomy [2], and a Berlin EXCOR aortic cannula with an interposition graft sutured to the ascending aorta [3] as the outflow cannula. **B** LDH drop (asterisk) after the Berlin EXCOR cannula insertion as compared to the conventional central veno-arterial ECMO. ECMO, extracorporeal membrane oxygenation; LDH, lactate dehydrogenase

A 2-year-old, 13-kg female patient was diagnosed with idiopathic RCM at 15 months of age leading to end-stage heart disease. Echocardiography showed a non-dilated left ventricle with a 50% ejection fraction and a severely enlarged left atrium (Fig. 1A). The patient had multiple heart failure exacerbations and a near cardiac arrest. There was no pulmonary hypertension by cardiac catheterization. The patient was listed for cardiac transplantation. While listed, the patient had a heart failure exacerbation from an Enterovirus respiratory tract infection needing intravenous milrinone. The patient improved after 3 weeks and was weaned off milrinone. The patient had a sudden unexpected cardiac arrest as a step-down patient. The cardiac arrest was refractory to cardiopulmonary resuscitation (CPR) and required extracorporeal CPR. Initial attempt to cannulate via the right neck vessels was unsuccessful due to bleeding from a central line in the jugular vein. Hence, an emergent sternotomy and central cannulation of the ascending aorta and right atrium for veno-arterial extracorporeal membrane oxygenation (VA-ECMO) was performed (Fig. 1B). The cannulation time was 15 min and time to reperfusion was 70 min due to mobilization of the on-call team from home. Post-ECMO cannulation, there was severe biventricular failure and pulmonary hemorrhage with opacification of the lung fields on the chest X-ray (Fig. 1B, C, D, E). A left-ventricular vent was placed via the right superior pulmonary vein but thrombosed 12 h post-insertion (Fig. 1B). A percutaneous atrial septostomy was unsuccessful due to a thick atrial septum. The patient was neurologically preserved, following verbal commands and a head computed tomogram was unremarkable. Hence, a decision was made to convert the ECMO to a more stable mechanical circulatory support and perform a surgical septectomy for left heart decompression. With severe biventricular failure and pulmonary hemorrhage, the patient would require biventricular mechanical circulatory support and an oxygenator for respiratory support. Given the non-dilated ventricles, the drainage cannula would need to be atrial. On cardiopulmonary bypass (CPB) and cardioplegic arrest, an atrial septectomy was performed. A 6-mm Berlin heart EXCOR® aortic cannula (Berlin Heart, Berlin, Germany) was anastomosed to the ascending aorta with a 10-mm Hemashield (Getinge, Goteborg, Sweden) interposition graft (Figs. 1E, 2A(3)) and a 9-mm Berlin heart EXCOR® aortic cannula with a 20-mm GOR-TEX® (Gore, Newark, DE) interposition graft was anastomosed to the right atrial free wall (Figs. 1D, 2A(1)). The cannulas were sized by body surface area and the interposition graft by the cannula flange size. The cannulas exited the mediastinum by tunneling through the anterior abdominal wall and connected to the VA-ECMO circuit. The patient was transitioned from CPB to the Berlin VA-ECMO (B-ECMO) support with flows of 100–120 cc/kg/min. The chest was closed in the usual fashion after thorough mediastinal hemostasis. The patient was therapeutically anticoagulated with unfractionated heparin with an anti-Xa goal of 0.5–0.7 IU/m. The pulmonary hemorrhage gradually resolved by pulmonary toileting with dornase alfa (Fig. 1F, G). The patient was well supported on the B-ECMO with low hemolysis as indicated by the lactate dehydrogenase (LDH) levels as compared to higher levels on the central VA-ECMO circuit (Fig. 2B(asterisk)). The oxygenator was changed electively twice during the 40 days of support due to fall in the oxygenator partial pressure of oxygen (PaO_2_). The patient was extubated 10 days after pulmonary hemorrhage resolution, started on a nasogastric enteral diet, and participated in physical therapy. The patient successfully transplanted after 40 days of support with an uneventful postoperative course and was discharged (Fig. 1H).

## Discussion

Mechanically supporting RCM can be challenging. Left ventricle apical cannulation is suboptimal due to the small ventricular cavity size effectuating cavitary collapse around the cannula causing obstruction and clot formation [1, 2]. Also, apical cannulation may inadequately decompress the left heart causing persistent pulmonary congestion [2]. Direct left atrial cannulation is fraught with thrombus formation and collapse of the redundant left atrial tissue around the cannula [1, 4]. The limited ability to mechanically support RCM is reflected by the fact that only 2% of the pediatric mechanical circulatory support were of the RCM type [3]. To circumvent these issues, an indirect left atrial cannulation technique via a trans-right atrial approach through an interposition graft sutured to a surgically created atrial septal defect has been described [1, 4]. This avoids an intra-atrial cannula and preventing left atrial thrombus formation [1]. However, this technique can be challenging when biventricular support is needed as the right atrial cannula may be challenging to place with the indirect left atrial cannula occupying the space.

RCM with biventricular failure, use of paracorporeal support such as the Berlin heart, and need for pre-implant ECMO have a high mortality of 50–60% [1, 3]. A significant proportion of mortality is from infection especially with paracorporeal support devices [1, 3, 5]. With the need for four cannulas exiting the patient needing biventricular support, the risk for infection due to track site can increase, especially in small patients. To circumvent these issues, we simplified the mechanical support in our patient. Hence, the right atrial free wall was instead used to drain both the atria after the septectomy. By suturing a Berlin cannula with an interposition graft, there was no intra-atrial cannula, obviating the risk fo6r thrombus formation and collapse of atrial tissue around the cannula. The outflow was an aortic cannula on the ascending aorta. The Berlin cannulas were connected to a VA-ECMO circuit. With only two cannulas and the chest closed, this configuration provided a stable cannulation with cardiac and respiratory support. The Berlin aortic cannula is an 18-Fr (6 mm) cannula as compared to the 14-Fr aortic central ECMO cannula. Similarly, the atrial cannula is a 27-Fr (9 mm) cannula as compared to the 18-Fr venous central ECMO cannula. The benefit of larger Berlin cannulas was evident by the drop in the LDH (a marker of hemolysis) due to low turbulence as compared to the central VA-ECMO cannulas (Fig. 2B). This helped with a lowered risk of hepatorenal injury due to lower risk of hemolysis [6]. Similarly, platelet transfusion secondary to destruction as in conventional ECMO was not needed [6]. With the chest closed, the risk of infection and bleeding was minimized. A similar technique using the Berlin cannula with both veno-venous (VV) and VA-ECMO but for pulmonary failure in patients awaiting lung transplantation with a median support of 56 days has been described [7]. However, here with VA-ECMO support, main pulmonary artery drainage and aortic outflow was used as the goal was right heart and pulmonary support. With stable cannulation provided by the Berlin cannula and the chest closed, the patients can be extubated, mobilized, and participate in physical therapy awaiting a transplant. The downside of using VA-ECMO is the need to change the ECMO circuit due to a decrease in the efficiency of the oxygenator causing exposure to blood products and potential for sensitization. However, this was not an issue in our case or in patients undergoing the lung transplant [7]. An oxygenator has been effectively temporarily intercalated with biventricular Berlin heart EXCOR® device, either on the right or left ventricle assist side until lung recovery and would have been alternative strategy in this case [8, 9]. However, these patients were of the dilated cardiomyopathy type with a more favorable cannulation options such as cannulation of the left ventricular apex unlike the RCM type with suboptimal biventricular cannulation options. In addition, the pulmonary failure was of pneumonic type with shorter oxygenator use only for a few days and the success of bridge to a heart transplant was variable despite the adequate support due to sepsis.

## Conclusion

Mechanically supporting RCM as a bridge to transplant can be challenging especially with biventricular and pulmonary failure. The use of Berlin cannula with a modified uniatrial drainage and VA-ECMO can simplify the support.

## Data Availability

Not applicable, case report.
